# Left Atrial Structural Remodelling in Non-Valvular Atrial Fibrillation: What Have We Learnt from CMR?

**DOI:** 10.3390/diagnostics10030137

**Published:** 2020-03-02

**Authors:** Mariana Floria, Smaranda Radu, Evelina Maria Gosav, Dragos Cozma, Ovidiu Mitu, Anca Ouatu, Daniela Maria Tanase, Viorel Scripcariu, Lacramioara Ionela Serban

**Affiliations:** 1 Department of Cardiology, Emergency Military Clinical Hospital, 7-9 General Henri Mathias Berthelot Street, 700483 Iasi, Romania; floria_mariana@yahoo.com; 2 Faculty of General Medicine, Grigore T. Popa University of Medicine and Pharmacy, 16 University Street, 700115 Iasi, Romania; dr.evelinagosav@yahoo.com (E.M.G.); mituovidiu@yahoo.co.uk (O.M.); ank_mihailescu@yahoo.com (A.O.); tanasedm@gmail.com (D.M.T.); viorel.scripcariu@umfiasi.ro (V.S.); ionelaserban@umfiasi.ro (L.I.S.); 3 Department of Cardiology, Cardiology Clinic, “Prof. Dr. George I.M. Georgescu” Institute of Cardiovascular Diseases, 700503 Iasi, Romania; 4 Department of Internal Medicine, IIIrd Medical Clinic, “Sf. Spiridon” Emergency Hospital, 1 Independentei Street, 700111 Iasi, Romania; 5 Department of Cardiology, Victor Babeș University of Medicine and Pharmacy, 300041 Timișoara, Romania; dragoscozma@gmail.com; 6 Department of Cardiology, Cardiology Clinic, “Sf. Spiridon” Emergency Hospital, 1 Independentei Street, 700111 Iasi, Romania; 7 Department of Surgery, Regional Institute of Oncology, 2-4 General Henri Mathias Berthelot Street, 700483 Iasi, Romania; 8 Department of Physiology, “Grigore T. Popa” University of Medicine and Pharmacy, 16 University Street, 700115 Iasi, Romania

**Keywords:** atrial fibrillation, atrial fibrosis, structural remodeling, cardiac magnetic resonance

## Abstract

Left atrial structural, functional and electrical remodelling are linked to atrial fibrillation (AF) pathophysiology and mirror the phrase “*AF begets AF*”. A structurally remodelled left atrium (LA) is fibrotic, dysfunctional and enlarged. Fibrosis is the hallmark of LA structural remodelling and is associated with increased risk of stroke, heart failure development and/or progression and poorer catheter ablation outcomes with increased recurrence rates. Moreover, increased atrial fibrosis has been associated with higher rates of stroke even in sinus-rhythm individuals. As such, properly assessing the fibrotic atrial cardiomyopathy in AF patients becomes necessary. In this respect, late-gadolinium enhancement cardiac magnetic resonance (LGE-CMR) imaging is the gold standard in imaging myocardial fibrosis. LA structural remodelling extension offers both diagnostic and prognostic information and influences therapeutic choices. LGE-CMR scans can be used before the procedure to better select candidates and to aid in choosing the ablation technique, during the procedure (full CMR-guided ablations) and after the ablation (to assess the ablation scar). This review focuses on imaging several LA structural remodelling CMR parameters, including size, shape and fibrosis (both extension and architecture) and their impact on procedure outcomes, recurrence risk, as well as their utility in relation to the index procedure timing.

## 1. Introduction

Atrial fibrillation (AF) is the most frequent cardiac arrhythmia and is associated with increased risk of stroke, mortality and decreased quality of life [1]. Left atrial (LA) structural, functional and electrical remodelling is linked to AF pathophysiology and mirror the phrase *“atrial fibrillation begets atrial fibrillation”* [2]. The different types of atrial remodelling are interconnected, as structural remodelling leads to LA dysfunction and subsequent electrical changes in the cardiomyocytes [2]. Fibrosis is the hallmark of LA structural remodelling and is associated with increased risk of stroke, heart failure (HF) development and/or progression and poorer catheter ablation (CA) outcomes with increased recurrence rates [3,4]. Subsequently, imaging structural remodelling is necessary given its impact on CA candidate selection, technique and post-procedural outcomes and prognosis. Cardiac magnetic resonance with late-gadolinium enhancement (CMR-LGE) is the gold standard in imaging fibrosis [5], however, it is not widely available and different centres failed to reach an agreement regarding scanning protocols. We will further analyse the utility of various CMR-derived imaging parameters of LA structural remodelling, including size, shape and fibrosis (both extension and architecture) in relation to the index ablation procedure timing in non-valvular AF patients in terms of candidate selection, ablation strategy and post-procedural outcomes.

## 2. Atrial Cardiomyopathy and Left Atrial Remodelling

LA is a thin-walled structure of varying thickness (1 to 15 mm), postero-superior to the right atrium with its four pulmonary veins (PVs) located postero-superiorly in a dome-like shape [5]. The left atrial appendage (LAA) is narrower than that of the right atrium with over 90% of the thrombi of AF patients forming at this level. [1,5]. Its morphology varies with non-chicken wing morphology being associated with increased thromboembolic risk [2].

The importance of LA function resides in its contribution with nearly 30% to the ventricular stroke volume [5]. LA behaves like a reservoir during ventricular systole, a conduit in early ventricular diastole and as a booster pump in late systole. Subsequently, its dysfunction has been associated with increased risk of stroke [6], poorer ablation outcomes and overall prognosis [7].

LA remodelling can be defined as the time-dependent structural, functional and/or electrical alterations in response to mechanical (pressure and/or volume overload), metabolic or electrical stressors, being the substrate for veritable atrial cardiomyopathy [2,6]. Initially reversible (<1 week of exposure) and adaptive, in time, the cellular, electrical and autonomic nervous alterations (Table 1) will become permanent and maladaptive [2].

Several conditions including heart failure, arterial hypertension, and valvular heart disease promote atrial remodelling through either pressure and/or volume overload. Atrial arrhythmias, especially AF alter the atrial structure, leading to irreversible changes in shape and function [5,6]. Moreover, the aforementioned diseases promote AF through LA remodelling (‘*AF begets AF*’).

The different types of LA remodelling (structural, functional, electrical) are interconnected [2], influencing both therapeutic options and prognosis. Fibrosis associated with structural remodelling leads to conduction heterogenicity, promoting re-entry and abnormal foci [6]. Furthermore, low-voltage areas correlate with fibrotic regions in AF patients [8] and LGE-CMR fibrotic burden is linked to LA dysfunction [2]. However, fibrosis and associated dysfunction may appear early during remodelling, preceding chamber enlargement [2]. They are linked to an increased risk of stroke even in non-AF patients [8,9].

While in 2016, the European Society of Cardiology’s consensus on atrial cardiomyopathies defined this notion as either structural, functional and/or electrical atrial cardiomyocyte changes leading to clinically relevant symptoms [5]. More recently, Bisbal et al. defines atrial remodelling, atrial cardiomyopathy and atrial failure as three tightly interconnected, however distinct entities with underlying subtle differences [10]. The authors considered atrial remodelling as the initial cardiomyocyte response to various stressors (pressure and/or volume overload, arrhythmias), such as electrical and structural remodelling in response to repeated arrhythmic events (atrial fibrillation) with resulting changes in atrial geometry (size and sphericity), function and electrophysiology. Atrial cardiomyopathy translates into a diseased and fibrotic myocardium, with a subsequent risk of developing heart failure and atrial dysfunction [11,12,13]. The previously attempted classifications of atrial cardiomyopathies are, however, rarely used in clinical practice, since it is mostly histological-based and hard to implement in day-to-day clinical practice [5,11]. Whether the atrial cardiomyopathy is primary (i.e., idiopathic atrial cardiomyopathy) or secondary to various pathologies, the underlying fibrotic burden is associated with atrial dysfunction and arrhythmias. Furthermore, there are still underdiagnosed conditions that may lead to atrial cardiomyopathies and arrhythmias, such as atrial ischemia and myocarditis with atrial-involvement [10]. Similarly, Guichard et al. focused on defining atrial cardiomyopathies and emphasized that the atrial remodelling leads, in fact, to the development of atrial cardiomyopathy [11].

Interestingly, the authors introduce atrial failure as ”any atrial dysfunction causing impaired heart performance and symptoms […] in the absence of significant valvular or ventricular abnormalities”. Even more interesting is the example that the authors offer: that of a lone AF CHA_2_DS_2_-VASc 0 patient with increased atrial fibrotic burden [10].

**Figure 1:**
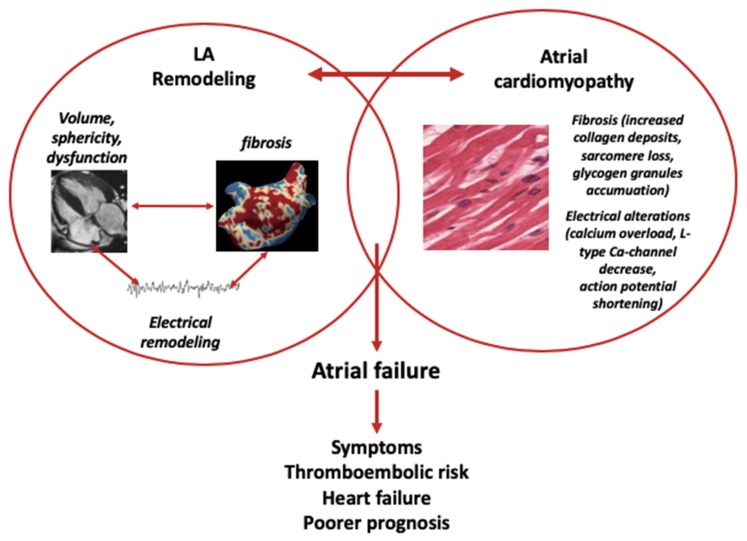
The bidirectional relationship between LA remodeling, atrial cardiomyopathy and atrial failure.

The three entities are interconnected and should be regarded as a pathophysiological continuum, with LA remodelling leading to/being a result of atrial cardiomyopathy, with relevant symptoms being a sign of progression towards atrial failure (Figure 1). Moreover, atrial failure is associated with increased thromboembolic risk, highlighting the importance of LA in thrombogenesis and the hypothesis that the increased thromboembolic risk seen in AF patients might, in fact, be mostly due to the underlying atrial failure rather than the AF per se.

Given the therapeutic and prognostic implications of LA remodelling and the subsequent atrial cardiomyopathy in AF patients, properly identifying it becomes necessary. As such, we will further review LA structural remodelling with the available imaging techniques and its utility in routine clinical practice.

## 3. Left Atrial Structural Remodeling

The hallmark of LA structural remodelling is myocardial fibrosis [2], atrial enlargement being the final expression of the latter. It has been shown that six weeks of AF increase fibrosis amounts [6]. From this perspective, some authors have highlighted fibrotic atrial cardiomyopathy as being the substrate for AF maintenance and/or progression and its increased risk of thromboembolic events [8].

Until recently, atrial fibrosis was regarded as a consequence of AF, but studies have shown that increased amounts precede and contribute to AF development in sinus rhythm patients [6,7,14]. This can be explained by the fibrotic effect of several comorbidities regarded as AF risk factors (i.e., arterial hypertension, heart failure, diabetes) [2]. At a molecular level, this is supported by the pro-fibrotic effects of angiotensin II, aldosterone, TGF-β1 and pro-inflammatory cytokines and the reported reverse-remodelling following therapy with either aldosterone receptor blockers or angiotensin-converting enzyme inhibitors [2].

In time, a fibrotic LA progresses to overt enlargement. The latter is the most easily imaged facet of structural remodelling (however, chronologically the last), as echocardiography is readily available and there are many firmer agreements on the recommended parameters [15]. Recently, there has been a shift from focusing on LA size to LA shape, as it is known that its dilatation is asymmetrical in the beginning, progressing from a discoid shape towards a sphere [16]. Echocardiographic or CMR-derived sphericity index seems to predict post-ablation recurrence rates and overall prognosis [16,17,18,19].

Different imaging techniques including echocardiography, computer tomography (CT) and CMR have been used to assess structural remodelling. This is useful in developing a patient-tailored approach for AF ablation as it may improve candidate selection, influence ablation strategies and determine prognosis. However, incorporating the results into clinical decision-making tools remains a challenge mainly because there is yet a universal definition of both LA structural remodelling and reverse-remodelling.

### 3.1. Left Atrial Size

It is agreed upon that AF patients have increased LA dimensions and these are associated with a poor overall prognosis, including poorer ablation outcomes and increased recurrence rates [1]. From this perspective, LA is currently being regarded as a veritable biomarker predictive of cardiovascular and thromboembolic events even in non-AF patients [2].

LA diameter underestimates its dimensions, as such, it is no longer recommended in assessing enlargement [1,15]. Although increased antero-posterior diameter is associated with post-CA recurrences, LA dilatation is asymmetrical and preferentially occurs in two directions: medial-lateral and supero-inferior [20]. It follows that assessing LA dimensions by diameter is inaccurate and LA volume (LAV) is preferred [15]. The European Society of Cardiology (ESC) considers an indexed LAV of >34 mL/m^2^ indicative of an enlarged LA [15]. Although routinely evaluated through echocardiography, CMR is the gold-standard in chamber dimensions quantification.

A recent meta-analysis including 21 studies and a total of 3822 patients assessed the relation between LAV and AF recurrence [20]. The authors concluded that patients with recurrences had higher LAV and indexed LAV (LAVI). Moreover, both emerged as independent predictors [20]. For each 1 mL increase in LAV/LAVI, there was a 3% increase in AF recurrence risk while a 1.84 mm increase in diameter was equivalent to a 0.8 mL increase in LAV. In addition, increased LA dimensions predicted AF development. Habibi et al. showed that increased LAV was an independent predictor of AF in a asymptomatic population [7] and that a 5 mm increase in LA diameter nearly doubled the risk of AF. Not only this, but increased dimensions are associated with LA dysfunction and increased risk of stroke independent of AF and CHA_2_DS_2_-VASc risk score [15,20,21].

### 3.2. Left Atrial Shape

Given the asymmetrical pattern of LA dilatation, several authors have focused on the LA shape. It seems that while dilating, LA’s shape evolves towards a sphere [17,18,19,20]. This geometrical structural remodelling is also associated with poorer ablation outcomes and recurrences [20].

The sphericity index compares the LA shape as determined by 3D CMR to a sphere and expresses this similarity through a percentage. It seems that patients with higher sphericity indexes have an 11 times higher risk of developing 1-year post-CA recurrences as compared to those with discoid LA [22]. In the LAGO-AF study, the LA sphericity index emerged as the sole independent predictor of recurrences [22,23,24]. Subsequently, geometrical structural remodelling may be superior to size in predicting CA outcomes and arrhythmia recurrences. Moreover, persistent AF patients tend to have a more spherical LA [17] and increased baseline sphericity index is associated with poorer ablation outcomes.

In a study conducted by Moon et al., a sphericity index of >0.87 strongly correlated with increased recurrences and weakly with LAV [19].

### 3.3. Left Atrial Fibrosis

Late-gadolinium enhancement CMR (LGE-CMR) is the gold standard in assessing myocardial fibrosis [15,23]. A gadolinium-based contrast agent enhances fibrosis detection due to its accumulation in the extracellular space (increased in fibrotic tissues) and its delayed clearance. This strengthens the T1 weighted signal due to gadolinium’s paramagnetic properties [8]. Images are acquired 15 to 20 min after contrast agent administration; however, adjustments are made based on injected amounts and the patient’s renal function.

The most widely used LGE-MRI fibrosis scan protocol is the DECAAF protocol [9]. Done on either 1.5 or 3 Tesla MRI scanners, the overall scanning time is approximately 15 min, based on detected artefacts and underlying rhythm.

The high-resolution LA images are initially acquired 15–30 min after gadolinium-based contrast agent administration by delivering an ECG gated and respiratory-navigated (3D inversion-recovery gradient echo pulse. ECG gating implies synchronizing image acquisition to a certain cardiac cycle phase (atrial diastole), with the purpose of minimizing motion artefacts and maximizing the image acquisition window [25]. When assessing atrial fibrosis, image acquisition is preferably done at the end of the expiration and in atrial diastole. In this regard, the patient’s underlying heart rhythm may be an issue. The image acquisition is triggered shortly after the beginning of the QRS complexes (the beginning of the atrial diastole) and is normally limited to below 20% of the RR intervals duration (maximum 200 ms).

In arrhythmic patients (either AF or frequent premature beats patients), it is recommended that the image acquisition is done shortly after R wave onset, within 10%–12% of the RR intervals duration. It has been emphasized that both heart rate and the rhythm’s regularity influence image quality, with heart rates >100 bpm leading to blurred images regardless of the underlying rhythm [26].

Fat saturation is conducted and after acquiring the cine-sequences used for the 3D anatomical reconstruction, the images are further processed. An operator manually defines the LA endocardial border, while excluding the mitral valve and left ventricle in order to define the LA segments. The heterogeneous image intensities are corrected before assessing fibrosis by using the blood pool as a comparator [25,26]. The software automatically/semi-automatically defines fibrotic versus non-fibrotic myocardium by detecting the subsequent difference in pixel intensity thresholds (normally, 2 to 4 SD). The operator inspects and adjusts for the possible inadequacies. It must be noted that this is an essential step and that the intensity thresholds vary across patients as they depend on various factors, including contrast agent concentration and time of image acquisition in relation to its administration and underlying heart rhythm. After 3D reconstruction, blue translates into normal myocardium, with green and yellow pointing out towards a fibrotic LA (however, colors may slightly vary across centers and software).

#### 3.3.1. Left Atrial Fibrosis as a Predictor of Post-Ablation Recurrences

After the CA scar in AF patients was identified on LGE-CMR scans [2,21], this technique was intensely studied for its ability to evaluate AF substrate and predict therapeutic response and post-ablation recurrence rates [2,3,6,27]. Oakes et al. showed that fibrosis extension correlated with increased arrhythmia recurrence rates at six months follow-up post-CA [21]. In a different study, patients with persistent AF and >35% LA fibrosis had increased recurrence rates [28]. Specifically, the recurrence risk was 1.5 higher for each 10% increase in the LA-LGE. Similarly, Marrouche et al. characterized AF patients fibrosis severity using Utah classification in stage I (<10%), stage II (10%–20%), stage III (20%–30%) and stage IV (>30%) (Figure 1) and reported a 6% recurrence risk for each 1% LA fibrosis increase [3].

In a recent multivariate analysis comparing the impact of different CMR-derived remodelling parameters (including LA volume, sphericity index, LA ejection fraction and fibrosis degree) on CA outcomes, only LA fibrosis emerged as a predictor of late AF recurrences (a median follow up of nearly seven years) [28]. On the other hand, LA volume and fibrosis do not always correlate, emphasizing the idea that a normally-sized LA could be extensively fibrotic [29,30].

It seems that the size of the largest fibrotic region is also important [27]. A different analysis of the DECAAF study revealed that the dimensions of the largest fibrosis patch predict recurrences in Utah stages II and III patients [31].

Interestingly, fibrosis disposition is inhomogeneous with its different locations being linked to arrhythmia recurrences. The posterior wall and left inferior pulmonary vein are preferentially affected, especially in persistent AF patients [32,33]. This is in accordance with histological studies [6]. Moreover, it seems that the posterior wall is the most affected irrespective of AF history. Consequently, a theory emerged that the increased wall stress at the level of the pulmonary veins and posterior wall (due to its proximity to the descending aorta) could in time lead to fibrosis and contribute to AF maintenance and/or post-ablation recurrence [33].

#### 3.3.2. Left Atrial Fibrosis and Thromboembolic Risk

The extension of baseline LA fibrosis also means higher thromboembolic risk [8,9,34] and subsequently, higher risk for developing major adverse cardio- and cerebrovascular events (MACCE) [35]. In a study conducted by King et al., baseline LA fibrosis reported as Utah stages correlated with both thromboembolic risk scores CHADS_2_ and CHA_2_DS_2_-VASc [35]. Patients in Utah IV (>35% LA fibrosis) had a higher incidence of MACCE (defined as either transient ischemic attacks, myocardial infarction, acute decompensated HF or CV death) and were four times more likely to develop a transient ischemic attack. Several studies also report that patients with increased LA fibrosis are most likely to have had sustained a stroke/transient ischemic attack [36] and that in the majority of them, transoesophageal echocardiography identifies an LA thrombus [37]. Moreover, the overall fibrosis extension had better c-statistics than both thromboembolic risk scores in predicting LAA thrombi (0.87 versus roughly 0.7) [36].

The presence of fibrotic atrial cardiomyopathy might explain the persistence of the increased thromboembolic risk even in patients maintaining sinus rhythm post-CA [35]. Notably, it seems that this risk remains constant even with long-term sinus rhythm maintenance.

The same fibrotic atrial cardiomyopathy correlated with embolic strokes of undetermined origin even in non-AF patients [9]. Patients with more than 12% fibrosis burden presented with strokes even in the absence of confirmed AF [9].

As such, anticoagulation might be an option even in sinus-rhythm patients with increased LA fibrosis and might justify continuing anticoagulation in patients with fibrotic LA who maintained post-ablation sinus rhythm. At a molecular level, this is supported by the pro-inflammatory environment and subsequent thrombogenic endothelial dysfunction found in fibrotic atria irrespective of the underlying rhythm [8]. In other words, blood stasis is not the only thrombogenic mechanism and fibrosis in itself may lead to higher thromboembolic risk.

Spronk et al. launched a different perspective, i.e., that hypercoagulability in itself may stimulate fibroblasts and increase fibrosis [34]. The fact that anticoagulating goats (nadroparin) resulted in decreased fibrosis could shift the current point of view regarding anticoagulation therapy from strictly preventing thromboembolic events to influencing the substrate by reducing fibrosis degree [34].

#### 3.3.3. Left Atrial Fibrosis and LA Dysfunction

There is a connection between LA dysfunction, LA fibrosis, increased thromboembolic risk and post-ablation recurrence rates. An altered reservoir function has been associated with both increased thromboembolic risk [38] and recurrence rates [39]. Another study emphasized the link between LA reservoir dysfunction (standard deviation time to peak strain) and increased thromboembolic risk [40]. The authors reported that adding parameters of mechanical LA dyssynchrony to the thromboembolic risk scores might change CHA_2_DS_2_-VASc c-statistics from 0.75 to 0.82 [40].

#### 3.3.4. Left Atrial Fibrosis and Heart Failure

Sustained AF leads to HF development and/or progression [1,2] and HF, in turn, is worsened in terms of prognosis and quality of life by the superposition of AF. It has been shown that AF patients with concomitant HF have increased LA fibrosis and subsequently, individuals with higher degrees of LA structural remodelling have lower baseline left ventricular ejection fraction (LVEF) [41]. Out of these, patients with less LGE extension benefit most from CA procedures in terms of LVEF improvement [41]. The mechanism proposed was that an intensely fibrotic LA is unable to contribute with the usual 10%–15% to the ventricular filling because it is stiffer and will not contract efficiently even if sinus rhythm is restored. Moreover, it seems that AF patients have increased LV fibrosis as determined by T1 weighed CMR scans, also contributing to the systolic dysfunction of these patients and worse prognosis [42].

The fact that AF is tightly linked from a pathophysiological point of view with HF in terms of development, impact on patients’ quality of life through symptoms worsening and overall prognosis support the use of CA to restore sinus rhythm in patients with reduced LVEF. The CASTLE-AF trial emphasized that HF patients with reduced LVEF and AF benefit in terms of survival and hospitalization rates from sinus rhythm restoration using CA procedures [43].

However, there is a delicate balance between risk and benefits in ablating AF HF patients. A very extensive ablation scar could, in turn, determine HF development and/or worsening [41]. Taking into consideration that a higher fibrotic LA would require additional substrate ablation (and, therefore, additional ablation lines) and that exactly these patients are more likely to have concomitant HF, ablating these patients becomes even more difficult. It is exactly for this reason that further studies are needed to determine an LA fibrosis threshold that would justify, on one hand, performing substrate ablation and on the other, exclusion from ablation procedures due to lack of symptomatic and prognostic benefit.

#### 3.3.5. Left Atrial Fibrosis and Electroanatomic Mapping

Another possible method for quantifying the atrial substrate and, indirectly, estimate the fibrotic burden, is three-dimensional electroanatomic mapping (EAM) systems bipolar voltage mapping, used in clinical practice to guide ablation procedures [44,45]. Low-voltage zones (LVZ) were arbitrarily defined as areas of a bipolar voltage of <0.5 mV, while silent areas (scars) translated into either no detection of electrical activity or a bipolar voltage of < 0.05 mV. Although several studies showed that patients presenting with increased LVZs (stages III and IV) present with increased post-ablation arrhythmic recurrences [44,46], there are several controversies regarding this technique.

First of all, the 0.5 mV threshold was arbitrarily chosen with no previous histological correlation. Despite this, conduction velocity in areas with a bipolar voltage between 0.5 mV and 1 mV seems to resemble that of the non-LVZs areas and fractioned electrograms were found exactly in these areas of <0.5 mV [44]. Moreover, these areas were associated with arrhythmia inducibility. Secondly, there has been no reported correlation between LVZs and histological specimens and the fact that no consensus is available between different techniques and catheters used makes comparison difficult across studies. Thirdly, the correlation between LGE-reported fibrosis and LVZs is controversial. While Oakes et al. reported that LGE correlated with LVZ defined as a bipolar voltage of <0.5 mV and provided histological evidence [21], Lim et al. point out that both techniques lack agreement in protocols and are difficult to compare [47]. Moreover, a mismatch has been reported between LGE and LVZ distribution within the LA [44]. While LGE was most frequently found in the posterior, lateral and inferior LA walls, LVZ was identified in the anterior wall, roof and interatrial septum [44]. Interestingly, in the study conducted by Platonov et al. evaluating histological LA fibrosis, the LA anterior wall and septum were not examined [48]. Moreover, a different study researching the association between EAM-identified LA rotors and LGE scans showed no correlation between the two [49].

Several causes might contribute to this discrepancy. First of all, the lack of agreement on the scanning protocols for both techniques may lead to heterogeneities and increased difficulties in comparing the two. Secondly, there are several technical challenges in determining LVZ, including electrode positioning in relation to the wavefront orientation, electrode spacing and its contact with the LA endocardium [44,50]. Difficult anatomical regions might result in undetectable LVZ caused by inappropriate contact. Determining both LVZ and LGE extension in patients while in AF is another challenge, as the arrhythmia both lowers the LGE image quality and increases motion artefacts and makes LVZ determination difficult by the varying directions of the wavefronts in relation to electrode positioning. Further studies are required to better compare the two techniques, as they are valuable assets in performing substrate and/or rotor-based AF ablation.

## 4. Left atrial Appendage Structural Remodelling

Although there is an agreement on the importance of LA remodelling in AF pathophysiology and patients’ management [51], there is a lack of consensus regarding the remodelling of LA appendage (LAA). The lack of this agreement is even more striking since almost 90% of the AF thrombi occur at this level [52,53].

The most studied parameter of LAA was its morphology, with a non-chicken wing type being associated with increased thromboembolic risk [54]. Khurram et al. revealed that while the morphology per se showed no correlation with thromboembolic risk, out of the LAA morphological parameters analysed, a more trabeculated LAA with a narrower orifice was associated with an increased risk of stroke, most likely due to increased blood stasis [54]. The authors highlighted that categorizing LAA through a pre-determined morphology is user-dependent and unreliable due to inter-observer variability. This, in turn, could account for the lack of correlations between LAA morphologies and thromboembolic risk across various studies.

Recently, authors focusing on LAA remodelling revealed higher post-ablation arrhythmia recurrence risk with each 1% increase in LAA fibrosis as assessed by LGE-CMR [55]. Moreover, the authors did not find a correlation between the degree of LA and LAA structural remodelling, most probably due to their different embryologic origin. Ma Nan et al. recently revealed that LAA fibrosis determined on histological specimens correlated with AF duration and post-ablation recurrence risk [56]. However, there are no studies reporting the direct correlation between LAA LGE extension and histological specimens.

The importance of assessing LAA remodelling lies beyond understanding AF pathophysiology; it might justify using additional ablation lines at this level. A different study showed that the LAA was the source of ectopic foci in 9% of the patients requiring a re-do procedure [57]. However, the authors highlight that this procedure may be associated with increased rates of cerebrovascular thromboembolic events and subsequent LAA thrombi formation [56].

## 5. Therapeutic Implications of CMR

### 5.1. Pre-ablation Fibrosis Assessment

**Figure 2:**
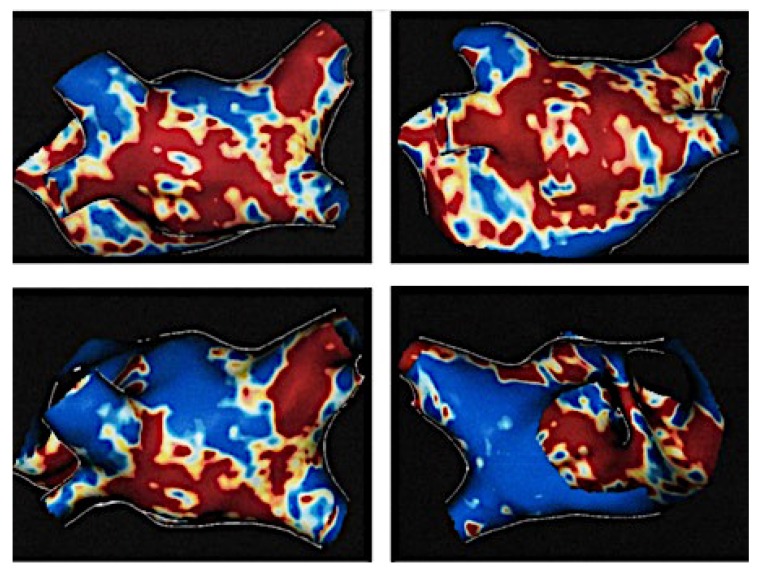
LGE-LA of 35.4% (Utah IV) in a persistent AF patient with repetitive ablations. Increased fibrosis at the posterior wall and pulmonary veins. Red demonstrates the presence of fibrosis. AF: atrial fibrillation; LGE-LA- late gadolinium enhancement in the left atrium.

Detecting and quantifying baseline LA fibrosis may alter therapeutic choices by influencing candidate selection and ablation strategy [8]. Excluding patients with advanced stages of diffuse fibrosis (Utah stage IV) and accepting those in Utah stages II–III [27] may be feasible (Figure 2).

Persistent AF patients show increased fibrosis and nearly half of the CA procedures either fail to restore sinus rhythm or are associated with recurrences [58]. It follows that they might benefit from substrate ablation in addition to classical PVI. The on-going DECAAF II trial will assess whether fibrosis ablation in addition to PVI will benefit persistent AF patients in terms of procedure success and arrhythmia-free survival interval [59].

Another proposed ablation strategy is using the 3D-LGE CMR models to predict and analyse electrophysiological behaviour of normal and fibrotic tissue [60,61]. Applying virtual pacing may help identify the re-entry drivers by observing the development of re-entry circuits in fibrotic regions. This offers a patient-tailored approach and several authors are researching the topic. Inhomogeneous LA fibrosis may call for different ablation strategies, such as posterior wall debulking or ablation of low voltage areas [32].

**Figure 3:**
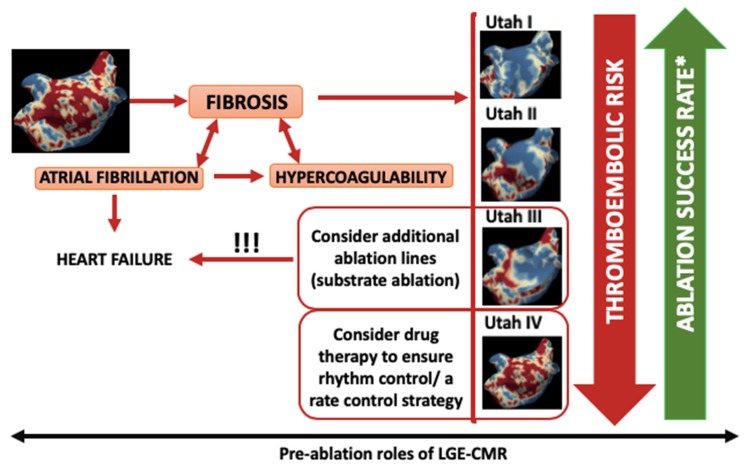
Pre-ablation roles of LGE-CMR.* in terms of immediate sinus rhythm restoration and post-procedural arrhythmia recurrences. LA: left atrium; LGE-CMR: late-gadolinium enhancement cardiac magnetic resonance, Utah classes of fibrosis: Utah I: <10%, Utah II: 10%–20%, Utah III: 20%–30%, Utah IV: >30%.

Figure 3 summarizes the pre-ablation roles of LGE-CMR in relation to both thromboembolic risk and procedural success rate.

### 5.2. Peri-Procedural CMR

Using real-time CMR to perform CA may offer several advantages over the conventional approach [28]. First, it minimizes radiation exposure for both medical professionals and patients. Second, it offers a real-time assessment of the anatomical structures, substrate, catheter position and ablation lesion (including gap areas during the index procedure). Pre-ablation electroanatomic mapping has indeed reduced exposure time; however, using a real-time CMR system would render fluoroscopy unnecessary [8,28]. The requirement of CMR-compatible equipment is being addressed by the development of new devices, such as CMR-compatible lasso catheters [62]. CMR-electrophysiological systems have been tested on animal models and recently 30 patients underwent atrial flutter CMR-guided ablation with satisfactory results [63]. However, peri-procedural CMR ablations have not yet been validated in AF patients. In addition, using such systems could be limited by the risk of gadolinium-induced nephropathy.

### 5.3. Post-Procedural CMR

#### 5.3.1. Assessing the Ablation Scar

Immediately post-ablation, myocardial oedema and localized necrosis limit CMR efficiency. As PV reconnection is one mechanism explaining AF recurrence, assessing ablation lesions becomes a priority. However, it is known that T1 weighted sequences and LGE cannot properly image the ablation scar immediately after the procedure [61]. Researchers have begun using T2 weighted sequences that can distinguish reversible myocardial oedema [61]. It seems that the adapted T2 sequence is superior to the dark-blood sequences in terms of image resolution and atrial wall border delineation. As such, it may be used with LGE scans to assess the ablation scar composition and help in indicating a re-do procedure [64].

LGE scans may be used to analyse ablation lesions gaps. Authors have highlighted that ablation scars show increased gadolinium uptake as compared to pre-existing fibrotic regions [8]. Other studies have reported gaps as dark no-reflow areas that better correlated with late post-ablation scans [61]. These post-ablation dark non-enhancing images correlated with no-reflow phenomena and have been shown to have a greater contribution to the formation of the permanent scar as opposed to hyper-enhancing lesions [64]. The latter reflects the ablated lesions, including both the scar and the surrounding post-procedural inflamed tissue. The importance of scar identification lies in its correlation with AF recurrences. An incomplete scar, with gap lesions, might even justify a re-do procedure in a symptomatic AF-recurrent patient.

These correlated with an electrical reconnection as determined by electro-anatomical mapping systems. However, there is room for improvement in CMR gaps detection; as there is still an issue regarding clinically relevant gaps that may justify a re-do procedure.

#### 5.3.2. Left Atrial Reverse Remodeling

Post-ablation, CMR scans can assess the degree of reverse-remodelling in response to sinus rhythm restoration [2,65]. Although there is no approved definition, it is considered that a 15% reduction of initial LAV (either echocardiographic or CMR-determined) mirrors the degree of reverse-remodelling [2]. LAV decreased post-sinus rhythm restoration during a seven year-period follow up [66]. Interestingly, patients who remained overweight and with left ventricular hypertrophy failed to reach the same degree of reverse remodelling, pointing out the importance of comorbidities management even post successful CA procedures [66]. At the same time, the sphericity index may be superior to volume in assessing reverse-remodelling [17].

A strong body of evidence supported LA as a type 0 biomarker in that its degree of structural remodelling correlated with the presence and/or progression of AF [2]. However, recent studies highlight the importance of evaluating LA reverse remodelling as a response to therapy. Authors have proven the prognostic abilities of LA remodelling even in non-AF patients, where an enlarged and/or fibrotic LA predicts cardiovascular events and even stroke [9]. Taking these into consideration, LA may now be considered either a type 1 or 2 biomarker instead of its historical type 0 classification [2]. A type 1 biomarker may be used in therapy follow-up while a type 2 biomarker may translate into clinical end-points and prognostic utilities [2].

#### 5.3.3. Post-Ablation Fibrosis Assessment

Post-ablation fibrosis has also proven a recurrence predictor [8]. Studies have shown that the formation of an ablation scar was associated with lower recurrences [8]. Patients with a scar burden of nearly ¼ of the LA wall volume showed decreased recurrence rates [6,8]. A proposed explanation is that increased ablation scar burden translates into less probable gaps, and therefore, fewer chances of incomplete electrical isolation. The ablation scar correlated on LGE scans with the low-voltage areas on electro-anatomical mapping systems and patients showing circumferential PV scars had fewer recurrences.

Residual fibrosis was defined as the unaddressed pre-ablation fibrosis during the index procedure. It has been reported that the amount of residual fibrosis at three months post-ablation is also associated with increased recurrences, which highlights the importance of additional substrate targeting in selected candidates [8].

Assessing LA fibrosis is essential in AF patients, however, there is an urgent need for common protocols across centres which will enable CMR scans comparison and longitudinal patient follow-up.

The contribution of CMR to LA structural remodelling assessment is summarized in Table 2.

#### 5.3.4. Post-Ablation Complications

Two of the most feared post-ablation major complications are pulmonary vein stenosis and atrio-esophageal fistula. Although they are both rare complications (under 6% reported incidence), they require extensive imaging since they are life-threatening (80% mortality in the case of atrio-esophageal fistula) [67].

PV stenosis may determine polymorphic symptomatology, ranging from asymptomatic to exertional dyspnoea, based on the stenosis severity (defined as >50% narrowing) [67]. Although best imaged through CT scans due to high spatial resolution, CMR may be an alternative due to the possibility of assessing both anatomy and PV hemodynamics [67,68,69,70,71]. Chang et al. showed that patients showing a post-procedural 20% PV narrowing tended to present with PV stenosis at three months follow-up [70]. Furthermore, the authors highlighted that patients with baseline narrower PVs assessed on pre-procedural CMR scans had a higher risk for post-procedural PV stenosis.

In the case of atrio-esophageal fistula suspicion, non-invasive imaging techniques may be considered as an initial diagnostic step, especially since upper gastrointestinal endoscopy should be cautiously indicated and performed due to higher risk of perforation [67]. Post-ablation CMR-scans reveal anterior esophageal enhancement, which usually self-resolves weeks after the procedure in the case of simple esophageal erosions. It may be reasonable to perform upper gastrointestinal endoscopy only in patients showing esophageal enhancement [67]. However, the authors report that this finding is quite frequent after the procedure (30%) [70].

**Figure 4:**
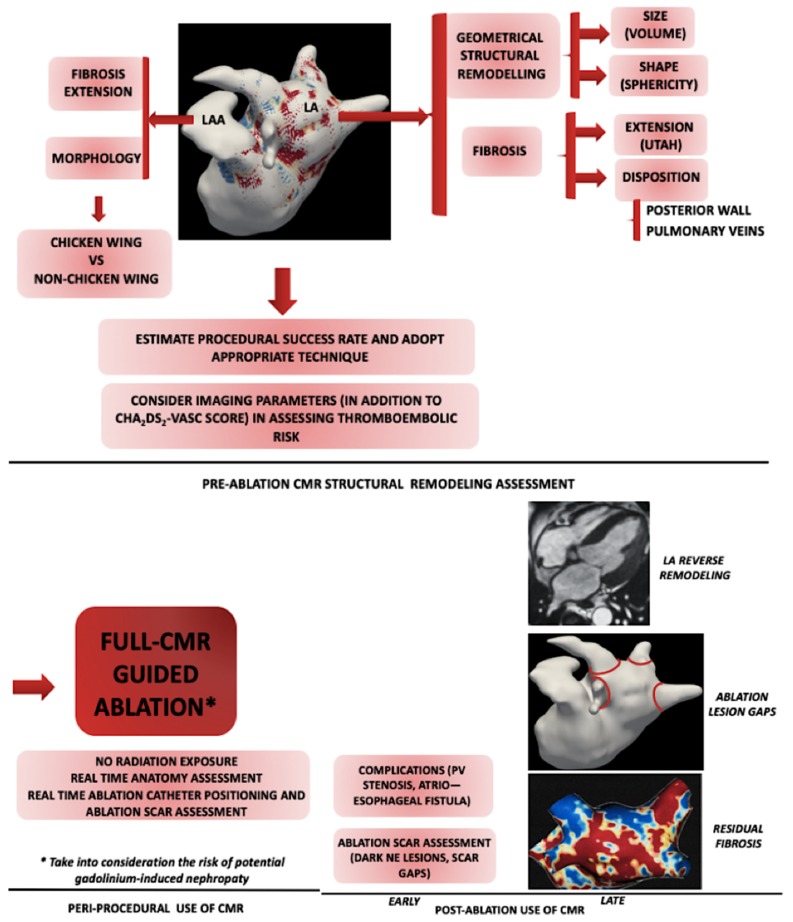
The use of CMR imaging in relation to the timing of the index procedure (up, center- pre-ablation, down-left- peri-procedural and down-right- post-ablation). Preablation, the degree of structural remodeling can be assessed through LA volume, sphericity and fibrosis extension and disposition. New techniques are being explored to allow performing full CMR-guided ablation. Post-ablation, CMR is used immediately after the procedure to scan for possible complications and assess early scar formation. Later, CMR can be used to assess the degree of both reverse remodeling and residual fibrosis and describe the ablation lesions gaps. CMR: cardiac magnetic resonance; LA: left atrium; LAA: left atrial appendage; NE: non-enhancing.

Figure 4 summarizes the roles of CMR in relation to the procedural timing.

## 6. CMR Limitations

However, it must be brought to attention that fibrosis quantification on CMR depends on several factors. Signal intensity may be influenced by gadolinium-based contrast agents dosing, image acquisition time in relation to contrast administration (proper inversion recovery time setting), a patient’s renal function, haematocrit, body mass index and underlying heart rhythm [8].

Manually tracing the LA wall border leads to error due to measurement subjectivity and inter- and intra-observer variability. Semi-automatic tracing software may erroneously delineate the thin LA wall (difficult in the context of limited spatial resolution). Additionally, the available scales and post-processing images techniques and software vary across centres [9].

The prolonged acquisition time may determine motion artefacts in patients with irregular heart rhythms or advanced pulmonary disease. In general, CMR scans are preferably ECG-triggered and performed under respiration gating. However, pulmonary disease patients are unable to hold their breath for a long time, which may result in motion artefacts.

It has been estimated that nearly ¼ of the LGE artefacts that made fibrosis assessment impossible was due to the presence of AF [51]. A possible solution would be attempting a temporary rhythm control strategy in order to improve image quality [51]. Another limitation of this imaging technique is claustrophobia (approximately 5% of patients being reported as claustrophobic) [72].

This lack of consensus, together with the scanning difficulties in various patients and the need for trained specialists have so far limited the use of CMR to clinical trials and tertiary centres. However, it is time to better explore the atrial cardiomyopathy in each AF patient [73] and CMR is the gold standard.

## 7. Conclusions

Structural remodelling is a complex manifestation of the underlying atrial cardiomyopathy, associated with an increased risk of developing AF even in healthy individuals. Properly imaging LA structural remodelling offers prognostic information and influences therapeutic choices. Determining candidate selection for catheter ablation and strategy by fibrosis extension and disposition seems reasonable. Researchers have already shown that substrate targeting in addition to classical PVI is beneficial in terms of ablation outcomes and arrhythmia-free survival interval in selected patients. Moreover, the development of CMR-guided ablation techniques may completely eradicate the current issue of radiation exposure. Using CMR to evaluate post-ablation lesions also shows promise in identifying gaps and justifying the need for a re-do procedure.

## Figures and Tables

**Table 1:** Cellular (mal) adaptive changes in left atrial remodelling.

Level	Change	Effects	Additional Remarks
Metabolic	Switch to fetal glycolysis (fatty acid beta-oxidation)	Reduced energy levels	−
Neuro- hormonal	Increased NPs, Ang II, aldosterone, TGF- β1 levels	Increased fibrosis	Ang II * + TGF−β1 ≥ fibroblasts ≥ increased collagen production;
Cellular	Fibroblast activation Fibroblast-to-myofibroblast differentiation	Increased fibrosis	Fibroblasts can conduct electrical impulses via connexins ≥ anisotropy and spontaneous phase 4 cardiomyocyte depolarization; Myofibroblasts are typical of a structurally abnormal myocardium
Electrical	↓ L-type Ca^2+^ current; ↑ K^+^ inward rectifier current I_K,Ach_ activation Abnormal gap junctions distribution	Reentry, AP shortening Atrial refractoriness shortening	Calcium overload promotes reentry through action potential shortening and membrane hyperpolarization

* Ang II induces cardiac fibrosis only in the presence of TGF-β1. Ang II: angiotensin II; NPs: natriuretic peptides; TGF-β1: transforming growth factor beta-1.

**Table 2:** Studies focusing on left atrial structural remodelling using cardiac magnetic resonance.

Authors, Year	Number of Patients	Type of Remodeling	Imaging Parameters *	Conclusion	Reference
Habibi et al. 2016	509	Size	LAV/LAVI	LAV predicts incident AF	[7]
Kriatselis et al. 2019	42	Size	LAV	Greater LA reverse remodeling in normoponderal patients	[66]
Bisbal et al. 2013	106	Size, shape	LAV, SI	Baseline sphericity predicts recurrences	[17]
Bisbal et al. 2014	102	Size, shape	LAV, SI	Baseline sphericity better than LAV in predicting recurrences	[18]
Nakamori et al. 2018	227	Size, shape	LAV/LAVI, SI	Baseline sphericity predicts recurrences	[16]
Oakes et al. 2009	81	Fibrosis	LGE	Fibrosis predicts recurrences Fibrosis correlates with low-voltage areas	[21]
Marrouche et al. 2014	272	Fibrosis	Utah	Fibrosis predicts recurrences	[9]
McGann et al. 2014	386	Fibrosis	% of LA wall LGE	Fibrosis predicts recurrences LGE- correlates with histological fibrosis	[65]
Habibi et al. 2015	90	Size, fibrosis	LAV, LGE	Fibrosis=dysfunction	[3]
Khurram et al.2016	165	Fibrosis	LGE	Fibrosis predicts recurrences, especially in persistent AF patients	[28]
Higuchi et al. 2018	160	Fibrosis	LGE extension in 6 LA segments	Fibrosis = inhomogeneous distribution; ↑ posterior wall and inferior PV antrum	[32]
Chrispin et al. 2017	179	Fibrosis, size	LGE, LAV	Weak fibrosis-LAV correlation;	[30]
Siebermair J et al. 2019	182	Fibrosis, size	Utah, LAV	LAV and obesity predicted fibrosis in non-AF patients	[51]
Chubb et al. 2019	89	Fibrosis, shape, size	LGE, LAV, SI, LAEF	Fibrosis and dysfunction predict recurrences	[29]

* To quantify LA fibrosis, both LGE extension and Utah classification are used across various studies. AF: atrial fibrillation; CMR: cardiac magnetic resonance; LA: left atrium; LAEF: left atrial ejection fraction; LAV: left atrial volume; LAVI: left atrial indexed volume; LGE: late-gadolinium enhancement; LASP: left atrial sphericity; LVEF: left ventricular ejection fraction; PVI: pulmonary vein isolation; SI: sphericity index.
